# Ultrasound-Assisted Extraction of Anthocyanins from *Malus* ‘Royalty’ Fruits: Optimization, Separation, and Antitumor Activity

**DOI:** 10.3390/molecules27134299

**Published:** 2022-07-04

**Authors:** Yixin Liu, Yuheng Zhao, Yue Zhuo, Yuwen Li, Jiaxin Meng, Yilin Wang, Houhua Li

**Affiliations:** 1 College of Landscape Architecture and Art, Northwest A & F University, Yangling 712100, China; lyx13426325830@126.com (Y.L.); wn15553803032@163.com (Y.Z.); zhuoyuegongzuo@163.com (Y.Z.); mengjiaxin26@163.com (J.M.); wangyilin97417@163.com (Y.W.); 2 Shanghai United International School, Gubei Secondary Campus, Shanghai 201103, China; ellaliyuwen@icloud.com

**Keywords:** *Malus* ‘Royalty’ fruits, anthocyanin, extraction, separation, antitumor activity, human gastric cancer

## Abstract

Red *Malus* ‘Royalty’ fruits are rich in anthocyanins. This study aimed to obtain the optimal parameters for the extraction and separation of anthocyanins from *Malus* ‘Royalty’ fruits and to evaluate the inhibitory effect of the enriched anthocyanin fraction on gastric cancer cells. Ultrasonic-assisted extraction was used for the extraction of the anthocyanins of Malus ‘Royalty’ fruit, and the extraction results showed that the optimum parameters were an extraction temperature of 20 °C, a solid–liquid ratio of 1:6 (g/mL), ethanol and formic acid contents of 70% and 0.4%, respectively, an extraction time of 40 min, and an ultrasonic power of 300 W. The optimum extraction parameters to achieve the highest anthocyanin yield by a single-factor experiment coupled with response surface methodology were identified. The separation results showed that the AB-8 macroporous resin was a better purifying material, with 60% ethanol as an adsorbent, and the adsorption–desorption equilibrium times were 6 h and 1 h, respectively. Cyanidin-3-galactoside was the main body composition separation of anthocyanins by a high-performance liquid chromatography-diode array detector. The antitumor activity results showed that the anthocyanins of *Malus* ‘Royalty’ fruits have a significant inhibitory effect on the gastric cancer cell line BGC-803. The in vitro cell viability test of CCK-8 showed that the inhibitory effect on tumor cells was more significant with the increased anthocyanin concentration, with a half maximal inhibitory concentration (IC_50_) value of 105.5 μg/mL. The cell morphology was observed by an inverted microscope, and it was found that the backbone of BGC-803 treated with a high concentration of anthocyanins was disintegrated and the nucleoplasm was concentrated. The mechanism of apoptosis was analyzed by Western blotting, and the results showed that with increasing anthocyanin concentration in the medium, the expression levels of the proapoptotic proteins Bax and Bak increased, and the expression levels of the antiapoptotic proteins Bcl-2 and Bcl-xL decreased, which coordinated the regulation of cell apoptosis. This research suggests that the enriched anthocyanin fraction from *Malus* ‘Royalty’ fruits have potential antitumor and adjuvant therapeutic effects on gastric cancer.

## 1. Introduction

Tumors are executioners that threaten human health. A tumor of the gastric epithelial mucosa is the site of gastric cancer and is a common malignant tumor [1]. With an estimated over one million new cases of gastric malignant tumors each year, it is the fifth most commonly diagnosed malignant tumor worldwide [2]. Recently, natural products with antitumor effects have attracted increasing attention such as plant polyphenols, flavonoids, polysaccharides, and anthocyanins [3,4].

*Malus* ‘Royalty’ fruits, whose pericarps and sarcocarps are red, belong to *Malus* (Rosaceae) and have abundant phenolic compounds [5]. Numerous studies have shown that the red fruits of *Malus* ‘Royalty’ are rich in anthocyanins [6]. As a natural antitumor product, anthocyanins have many advantages such as wide source availability, low cytotoxicity, and food safety [7]. Anthocyanins mainly contain cyanidin-3-galactoside and small amounts of cyanidin-3-*O*-glucoside and cyanidin-3-*O*-rutinoside, and they are water-soluble natural pigments that are widely found in plant organs [8].

Due to the unstable nature of anthocyanins, they are easily affected by factors such as temperature, extraction time, and solution pH during the extraction process. Therefore, the extraction of anthocyanins is challenging [9,10]. The current extraction methods are mainly solvent extraction, ultrasonic extraction, microwave-assisted extraction, and supercritical fluid extraction technology [11,12]. Compared to the traditional solvent extraction method, the ultrasonic-assisted extraction (UAE) method, which has the advantages of environmental friendliness, low cost, and high extraction efficiency, is a popular extraction method [13,14]. The extraction efficiency of various active compounds from *Malus* ‘Royalty’ fruit by the UAE method needs to be optimized. The extraction efficiency of anthocyanins is related to many parameters such as solvent mass fraction, extraction temperature, extraction time, and solvent type [15]. Hence, the single-factor parameters of anthocyanin extraction need to be optimized to maximize the extraction yield. Response surface methodology (RSM), which is a mathematical method that is applied to study the influence of multifactor interactions on the yield of target compounds, is also a key part of the extraction process [16]. The RSM was used to determine the optimal extraction conditions of anthocyanins under the interaction of multiple factors, which reduced the total number of experiments and saved time. Anthocyanins cannot be used in various fields because they are difficult to stably detect [17]. In the natural product purification field, column chromatography is used to separate and purify bioactive compounds. This method proved to be effective in many studies such as on Ficus carica and Chinese prickly ash [18,19]. This method was applied to *Malus* ‘Royalty’ fruits for the extraction and separation of anthocyanins in this study, which were the most basic and important parts of the research.

As an important polyphenolic substance, anthocyanin has a certain inhibitory effect on cancer and is a potential natural anticancer substance. Existing experiments have shown that the extracted red fruits of *Malus* are rich in anthocyanins and have an inhibitory effect on human gastric cancer cells [5,6]. However, the antitumor ability and mechanism of *Malus* fruit anthocyanins in vivo are unclear. Thus, the antitumor mechanism of anthocyanin in *Malus* fruits needs to be further explained and verified. The purpose of this research was to optimize the extraction conditions of anthocyanins from *Malus* ‘Royalty’ fruits using the ultrasonic-assisted extraction method, to obtain separated anthocyanins by AB-8 macroporous resin, to evaluate the antitumor activity of the enriched anthocyanins fraction obtained by the CCK-8 method, and to explain the mechanism of anthocyanin-induced apoptosis of BGC-803 cells by Western blotting analysis.

This study provides broader insights into the utilities and medical applications of *Malus* ‘Royalty’ fruits, which can act as a potential plant resource for natural pigments. The findings in this research are valuable to food technologists and manufacturers. *Malus* ‘Royalty’ fruits were also observed to possess anti-cancer functions, which lays the theoretical foundation for fruit market management and the processing of the fruit industry in future research and developments in this field.

## 2. Materials and Methods

### 2.1. Plant Materials

**Figure 1:**
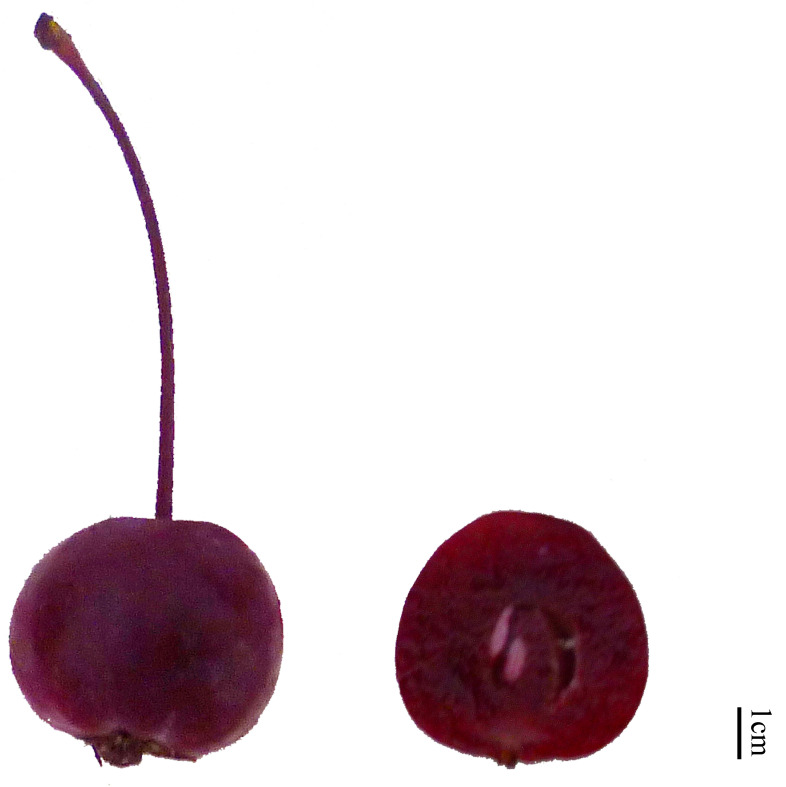
The morphology of *Malus* ‘Royalty’ fruits.

*Malus* ‘Royalty’ fruits were collected from the Germplasm Nursery of Northwest Agriculture and Forestry University, Yangling, China (Figure 1). The fruits’ average moisture content was 82.34%, and the average size among the samples was 2.2 cm in diameter. They were frozen at −80 °C for 48 h and freeze-dried in a vacuum for 72 h. After being crushed by a high-speed pulverizer, the materials were sieved with a 40-mesh screen before being stored in airtight containers until use.

### 2.2. The Reagents

AB-8 and X-5 macroporous resins were purchased from Tianjin Haoju Resin Chemical Factory (Tianjin, China). Amberlite XAD-7 macroporous resin, cyanidin-3-*O*-glucoside, cyanidin-3-galactoside, and cyanidin were provided by Shanghai Yuanye Biotech (Shanghai, China). Human gastric cancer cells (BGC-803) were obtained from Nanjing OGpharma Co. Ltd. (Nanjing, China). Paclitaxel was obtained from Sichuan Taiji Pharmaceutical Co. Ltd. (Chengdu, China). Other chemicals used in this study were of analytical grade.

### 2.3. Extraction and Determination of Total Anthocyanins

Freeze-dried powder of *Malus* ‘Royalty’ fruits (2.0 g) was mixed with different ethanol contents to obtain a certain solid–liquid ratio. The mixture was added to formic acid before extraction with a KQ-500DE ultrasonic instrument (Kunshan, China). The extract was then centrifuged at 6000× *g* for 15 min at 4 °C with a KDC-160HR centrifuge (Hefei, China). All experiments were performed three times under low light conditions.

The total anthocyanin content was determined by pH-differential spectrophotometry [20]. Briefly, 4.5 mL of two buffers (0.025 MKCl-HCl buffer, pH 1.0, and 0.4 M CH_3_COONa-HCl buffer, pH 4.5) was added to 0.5 mL of crude extracts. The mixture was stored in the dark for 15 min at room temperature. The absorbance of all samples was detected at 520 and 700 nm using a UV-2450 spectrophotometer (Shimadzu, Tokyo, Japan).

### 2.4. Ultrasonic Assisted Extraction Experiment (UAEE) Procedures

#### 2.4.1. Single-Factor Experiments

Choosing the right influencing factors and experimental scope helps increase the yield of anthocyanins. The main factors affecting the extraction efficiency include the extraction time, temperature, ultrasonic power, ethanol content, formic acid content, and solid–liquid ratio. The optimal parameters for these factors were evaluated when obtaining the maximum yield of anthocyanins by ultrasound-assisted extraction.

#### 2.4.2. Box–Behnken Design

Based on the single-factor test results, three factors that have the greatest impact on anthocyanin yield were screened out for the Box–Behnken design experiment [21]. Independent variables were selected as follows: extraction temperature (A, 10, 20, and 30 °C), ethanol content (B, 50, 60, and 70%), and solid–liquid ratio (C, 1:4, 1:5, and 1:6 g/mL). The optimal level was chosen as the center point of the designed experiment, and the number of total anthocyanins (Y) extracted was considered to be the response (dependent variable). The experimental plan shown in Table A1 generated a total of 17 test points.

### 2.5. Separation of Anthocyanins in Malus ‘Royalty’ Fruits

#### 2.5.1. Selection of Macroporous Resin

The macroporous resin was selected according to the method described by Ma et al. [22] with some modifications. The resin was treated with filter paper until dry, and then 2.0 g of the dried resin and 30 mL of the extracted solution were transferred to a conical flask. The flask was placed on a shaker (120 rpm) for 12 h until static adsorption equilibrium was reached. The absorbance of the supernatant was recorded at 520 nm, and the adsorption ratios of the resins were calculated according to Equation (1). In the desorption test, the three resins saturated with adsorption added 30 mL of 60% acidified ethanol solution (1% HCl). The mixture was treated as previously described. The resin desorption ratio was calculated by Equation (2).

(1) $$\mathrm{Adsorption}\;\mathrm{ratio}\;(\%)=\frac{\left(A_{1}-\;A_{2}\right)}{A_{1}}\;\times \;100\%$$

(2) $$\mathrm{Desorption}\;\mathrm{ratio}\;(\%)=\frac{A_{3}\;}{\left(A_{1}-\;A_{2}\right)}\;\times \;100\%$$

where A_1_ is the absorbance value of the solution before adsorption; A_2_ is the absorbance value after adsorption; and A_3_ is the absorbance value after desorption.

#### 2.5.2. Effect of Ethanol Content on Desorption Ratio

The selection of desorption agent on the three resins was as follows: 2.0 g samples of adsorbent saturated resins were added to different contents of ethanol–water–formic acid (20:79:1, 40:59:1, 60:39:1, 80:19:1, *v*/*v*/*v*). The flasks were placed on a shaker (120 rpm) for 60 min. The absorbance of each supernatant was recorded at 520 nm, and then the resin desorption ratio was calculated by Equation (2).

#### 2.5.3. Dynamic Purification Process

The column (30 × 600 mm) was wet packed with AB-8 macroporous resin, and 1–2 BV was eluted with deionized water to eliminate oxygen in the column [23]. The column was wrapped with tin foil to achieve the effect of light protection. The anthocyanin solution was uniformly added with a constant-current pump until the macroporous resin was adsorbed and saturated, and the column was eluted 1–2 BV with ultrapure water until the eluent was clear in color to remove the sugar and proteins. Then, 2–3 BV was eluted with 60% acidified ethanol, and the eluent was collected at a speed of 1 mL/min. One tube was collected per 50 mL. Full-wavelength scanning was performed on each tube to roughly determine the efflux of anthocyanins. When the effluent was colorless, the elution stopped, and the eluent was collected for high performance liquid chromatography (HPLC) identification and analysis.

### 2.6. HPLC-Diode Array Detector (HPLC-DAD) Analysis for Anthocyanin Components

The collected eluate sample was filtered through a 0.45 μm filter and detected for the composition and content using HPLC-DAD. The HPLC-DAD analysis was performed on a Shimadzu LC-2030C Liquid Chromatograph fitted with an Inertsil C-18 column (5.0 μm particle size, 4.6 × 250 mm). The mobile phases were A (0.04% formic acid–water mixture) and B (100% acetonitrile). The post-run time was set at 10 min as described by Han et al. [24]. The solution gradients were as follows: 0–40 min, 5–40% solvent B; 40–45 min, 40–100% solvent B; 45–60 min, 100% solvent B; and 60–70 min, 100–5% solvent B with a flow rate of 0.5 mL/min. Moreover, the column temperature, injection volume, and wavelength were set at 40 °C, 10 μL, 280 nm, and 520 nm, respectively.

According to our previous research [5] and the test results obtained from HPLC-DAD to quantify the anthocyanin obtained, the first drew the calibration curve for standard cyanidin-3-galactoside, and then we calculated the quantity of anthocyanin according to the standardized curve and the peak area with Equation (3), and the standardized curve is displayed as Supplementary Figure S1. The HPLC-analyzed target sample was concentrated by a rotary evaporator, the concentration was freeze-dried, and its anticancer activity was subsequently tested.

*P* (%) = (f_i_A_i_/**∑** f_i_A_n_) × 100% (3)

where *P* and A_i_ are the purity and peak area of the anthocyanins, respectively. A_n_ is the peak area of all phenols; f_i_ is the correction factor.

### 2.7. Antitumor Activity

#### 2.7.1. Cell Culture and Cell Viability

Human gastric cancer BGC-803 cells (1 × 10^5^ cells/well) were purchased from Nanjing OGpharma Co. Ltd and were cultured in Roswell Park Memorial Institute (RPMI-1640) medium containing 10% FBS, 100 μg/mL penicillin, and 50 μg/mL streptomycin. The cells were kept in a 5% CO_2_ humidified atmosphere at 37 °C, and the medium was replenished every two days.

The CCK-8 assay was performed as described in a previous study [5]. The separated anthocyanin powder was prepared in solution to the following concentrations: 0, 3.90625, 7.8125, 15.625, 31.25, 62.5, 125, 250, 500, 1000, and 2000 μg/mL. BGC-803 cells were treated with 100 μL of anthocyanin solution for 48 h, and the positive control was 10 μg/mL paclitaxel. After being cultured for 48 h, 10 μL of CCK-8 solution was added to each well and incubated for 4 h. Optical density was measured at 450 nm using a Thermo MK3 Enzyme-Linked Immunosorbent Assay (ELISA) reader. The anti-proliferative activities of separated anthocyanins are represented as half maximal inhibitory concentration IC_50_ values.

#### 2.7.2. Detection of Cell Morphology

To observe the morphological changes in BGC-803 gastric cancer cells, the cells (1 × 10^5^ cells/well) were plated in 96-well plates for overnight culture, and 100 μL of separated anthocyanins at different concentrations were added. Ten concentration gradients (0, 3.90625, 7.8125, 15.625, 31.25, 62.5, 125, 250, 500, 1000, and 2000 μg/mL) were made, and a blank group was included. After being treated for 48 h, the cells were rinsed twice with phosphate-buffered saline (PBS), and changes in cell morphology were observed on an XD-202 inverted fluorescence microscope.

#### 2.7.3. Apoptosis Assay

According to the experimental results of the CCK-8 assay, low, medium, and high concentrations for the apoptosis test were selected within the range of cell inhibition rates of 30, 50, and 60%. These cells were treated with 100 μL of separated anthocyanins at different concentrations (0, 20, 100, 200 μg/mL) for 48 h.

Apoptotic BGC-803 cells were analyzed using Annexin V-FITC/propidium iodide (PI). In brief, BGC-803 cells were washed twice with PBS buffer and suspended in 500 μL binding buffer (containing 5 μL Annexin V-FITC and 5 μL PI) after being treated with separated anthocyanins. The treated samples were left in the dark at room temperature for 10 min before determining the apoptosis rates using a BD FACSCalibur flow cytometer (OG pharma, Nanjing, China). The statistical numbers of V-FITC-positive and PI-positive cells in each field were analyzed.

#### 2.7.4. Cell Cycle Analysis

The expression of apoptosis-related proteins was detected by Western blotting according to the experimental method of Han et al. [5] (2019) with slight modifications. BGC-803 cells were treated with the enriched anthocyanin fraction at concentrations of 0, 20, 100, and 200 μg/mL for 48 h. Then, lysis was performed with lysis buffer (containing both protease inhibitors and phosphatase inhibitors) for 30 min. Next, loading buffer (1 × final concentration) was utilized to desaturate the protein for 10 min. Total protein (40 μg) was separated using a 12% polyacrylamide gel by electrophoresis and then transferred onto a nitrocellulose membrane. The membrane was blotted with 5% Bovine Serum Albumin (BSA) for 1 h after blotting and washed three times with Tris buffered saline with Tween-20 (TBST) before incubation with specific alkaline phosphatase-conjugated secondary antibodies for 1 h. Enhanced chemiluminescence reagent (P0018A, Beyotime) was used to identify an immunodominant protein marker, and pictures were taken immediately in the darkroom. The apoptosis-related proteins and glyceraldehyde-3-phosphate dehydrogenase (GAPDH) antibodies used in this experiment were provided by EnoGene™ (Nanjing, China).

### 2.8. Statistical Analysis

All data were obtained from three independent experiments, and the reported results were recorded as the mean ± SD. Data were analyzed using one-way analysis of variance (ANOVA). The RSM regression was analyzed by Design Expert V.10 software.

## 3. Results

### 3.1. Single-Factor Experiments for Anthocyanin Extraction

**Figure 2:**
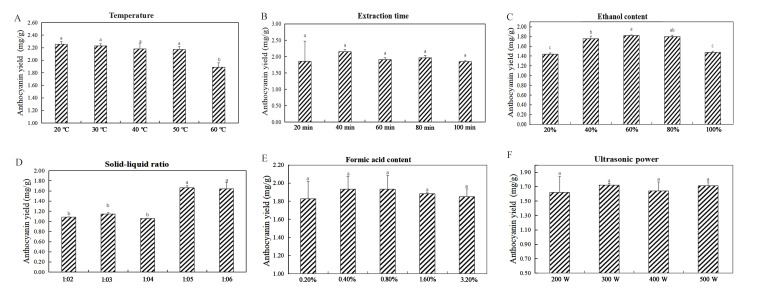
The influence of six single factors on anthocyanin extraction was tested. (**A**): Extraction temperature; (**B**): extraction time; (**C**): ethanol content; (**D**): solid–liquid ratio; (**E**): formic acid content; (**F**): ultrasonic power.

The influences of the six single factors on the extraction of anthocyanins were tested (Figure 2). The experiment showed that the extraction temperature had a significant effect on the yield of anthocyanins (*p* < 0.05). From 20 °C to 60 °C, anthocyanin production decreased with a gradual increase in temperature (Figure 2A). Therefore, temperature ranges of 10, 20, and 30 °C were selected for the RSM experiments as a reference. Experiments with different extraction times showed that the yield of anthocyanins markedly increased with increasing extraction time and reached the maximum value (2.151 ± 0.063 mg/g) at 40 min (Figure 2B). However, when the extraction time increased over 40 min, a decrease in the anthocyanin content was observed. Experiments with the ethanol content had a significant effect on the yield of anthocyanins (*p* < 0.05). As the ethanol content increased from 20% to 60%, the extraction yield increased, but the anthocyanin yield decreased when the ethanol content increased to 100% (Figure 2C). Therefore, ethanol content ranges of 50%, 60%, and 70% were selected for RSM experiments to provide a reference. The yield of anthocyanins differed in the experiments with different (*p* < 0.05) solid–liquid ratios (Figure 2D). The solid–liquid ratio increased from 1:2 g/mL to 1:5 g/mL as the anthocyanin yield increased and then slightly decreased when it increased to 1:6 g/mL. Therefore, solid–liquid ratio ranges of 1:4 g/mL, 1:5 g/mL, and 1:6 g/mL were selected for the RSM experiments to provide a reference. Experiments with different contents of formic acid showed that the anthocyanin yield was the highest (1.934 ± 0.139 mg/g) when the content of acid was 0.4%. With the increase in formic acid content, the anthocyanin yield gradually decreased (Figure 2E).

In summary, the pivotal influencing factors affecting extraction efficiency were ethanol content, solid–liquid ratio, and extraction temperature. Therefore, the experimental conditions were selected as follows: ethanol content from 50% to 70% (*v*/*v*), extraction temperature from 10 °C to 30 °C, and solid–liquid ratio from 1:4 to 1:6 g/mL for subsequent experiments.

### 3.2. Optimization of the Extraction Conditions

Through the single-factor experimental results, temperature, ethanol content, and solid–liquid ratio were selected as the reference factors of the response surface, and the variance analyses of the experimental results are shown in Table A2.

The order of the influence of various factors on the extraction of anthocyanins was ethanol content > solid–liquid ratio > extraction temperature. The experimental results showed that the linear coefficients of ethanol content (B < 0.0001) and solid–liquid ratio (C = 0.0014) were significant (*p* < 0.05). Additionally, the other two factors had a significant quadratic effect on the extractive efficiency (*p* < 0.05), except for B2. Among the interaction parameters, only AC affected the production of total anthocyanins (*p* < 0.05), and there was no notable influence on the others. Then, the experimental data were analyzed with multiple regression analysis, and the regression equation is shown in Equation (4):

Y = 1.58 + 0.055A + 0.2B − 0.12C − 0.016AB + 0.1AC + 0.012BC + 0.11A^2^ + 4.450E − 003B^2^ + 0.24C^2^ (4)

**Figure 3:**
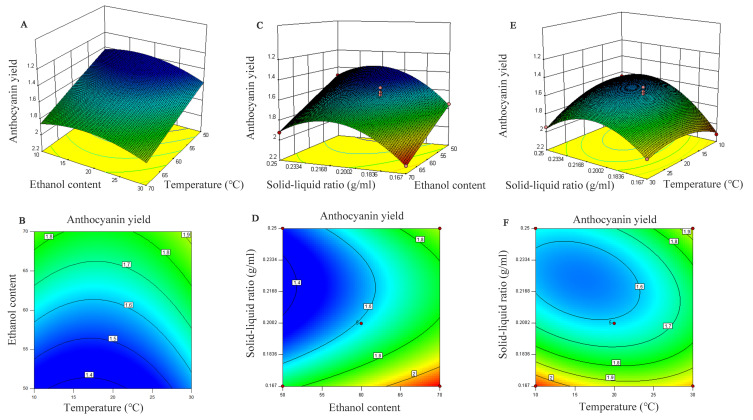
Three-dimensional response surface plots and corresponding contour plots. Influence of extraction ethanol content and temperature (**A**,**B**), solid–liquid ratio and ethanol content (**C**,**D**), and solid–liquid ratio and temperature (**E**,**F**) on anthocyanin yield from *Malus* ‘Royalty’ fruits.

The interaction between the solid–liquid ratio and extraction temperature had the most significant effect on the anthocyanin concentration (Figure 3). The regression analysis results were also consistent with the 3D response surface graph, and the p value of the interaction of these two single factors was less than 0.05.

According to the selected optimum conditions, the model scheme of the optimal response value was determined to be 70% ethanol content; extraction temperature, 20 °C; solid–liquid ratio, 1:6 g/mL; and Y was predicted to be 2.126 mg/g. Under these conditions, the actual measured anthocyanin yield reached 2.065 ± 0.015 mg/g, which was 97.13% of the predicted value, indicating that the model fits well with the actual situation.

### 3.3. Separation of Anthocyanins in Malus ‘Royalty’ Fruit

**Figure 4:**
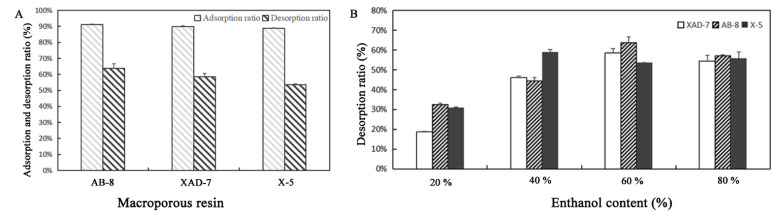
The selection of macroporous resin and eluant content. (**A**) Macroporous resin selection. (**B**) Desorption rate of resin under different ethanol concentrations.

The results of the resin selection test (Figure 4A) demonstrated that the adsorption capacities of the three resins were different: AB-8 > XAD-7 > X-5. The X-5 resin was nonpolar, and the XAD-7 and AB-8 resins were relatively weak in polarity. Therefore, the adsorption ratios of AB-8 and XAD-7 were better than that of X-5. The desorption capacities of the three resins were lower than the adsorption capacities: AB-8 > XAD-7 > X-5. After considering the adsorption and desorption ratio, AB-8 was selected as the final resin type for the purification experiment. 

The results of the eluant content test (Figure 4B) showed that as the ethanol content increased from 20% to 60%, the desorption ratio increased accordingly. The desorption capacity of ethanol on saturated resins significantly improved when the ethanol content reached 60% and the desorption ratio reached the maximum. However, when the ethanol content increased to 80%, the desorption ratio decreased. Thus, we selected 60% ethanol as an adsorbent for the dynamic desorption test.

The process of the dynamic elution experiment showed the change in anthocyanin content in each tube (Figure S2). In the full-wavelength spectral scanning diagram, the results showed that when approximately 100 mL of the eluent was collected, there was a clear absorption peak at 520 nm, indicating that anthocyanins began to flow out (Figure S3). When the eluent reached approximately 950 mL, absorption and desorption reached a balance and stabilized at the peak. The chromatographic profile of the separated sample at 525 nm was then obtained. The separation of the *Malus* ‘Royalty’ fruits’ total anthocyanins showed that the purity of the anthocyanin sample was 69.4%, and the major composition was cyanidin-3-galactoside.

### 3.4. Antitumor Activity

#### 3.4.1. The Effects of Different Concentrations of Anthocyanins on the Viability of BGC-803 Cells

**Figure 5:**
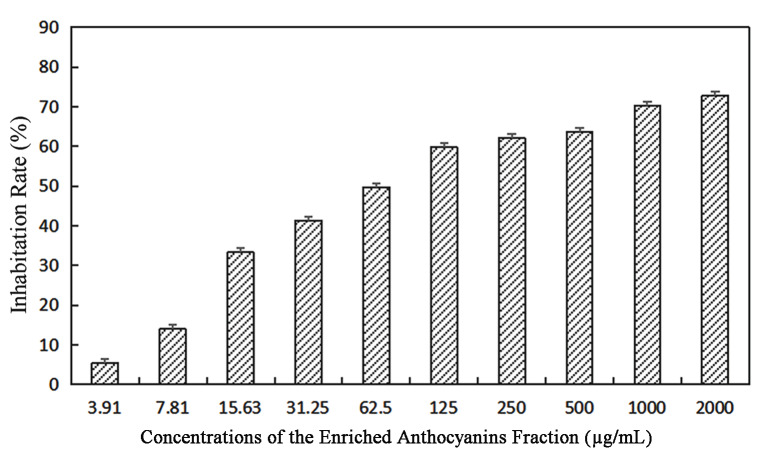
Effect of different concentrations of the enriched anthocyanins fraction on the viability of BGC-803 cells for 48 h (3.91–2000 µg/mL).

The inhibitory effects of the enriched anthocyanins fraction on BGC-803 gastric cancer cells were evaluated using the CCK-8 method. With increasing concentrations, the cell growth inhibition rates gradually increased and showed a dose-dependent phenomenon (Figure 5). When the concentration of the enriched anthocyanin fraction was 3.91–62.5 µg/mL, the inhibition was lower than 50%; when the concentration of anthocyanin was 2000 μg/mL, the inhibition rate reached 72.72%. The anthocyanins of the *Malus* ‘Royalty’ fruits had an appreciable inhibitory effect on BGC-803 gastric cancer cells, and the IC_50_ value was 105.5 μg/mL.

#### 3.4.2. The Effects of Different Concentrations of Anthocyanins on Cell BGC-803 Morphological Changes

**Figure 6:**
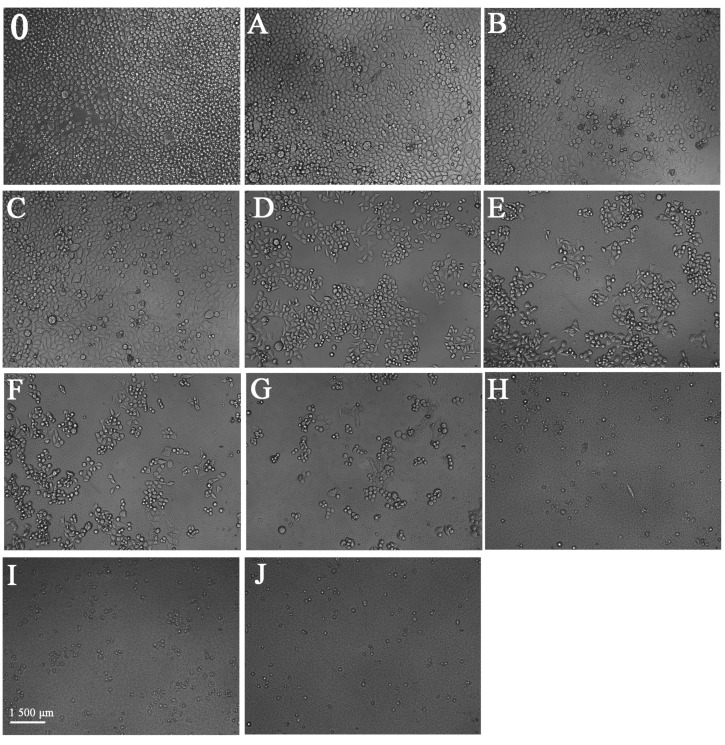
The cell morphological changes of BGC-803 cells after 48 h of anthocyanins treatment was observed by an inverted microscope. (**A**–**J**) represent anthocyanin concentrations of 3.906, 7.813, 15.63, 31.25, 62.5, 125, 250, 500, 1000 and 2000 μg/mL, respectively; (**0**) represents the blank group.

The morphological changes of BGC-803 cells treated with different concentrations of the enriched anthocyanin fraction for 48 h were evaluated (Figure 6A–J). Untreated cells without anthocyanins were complete and closely packed (Figure 6A-J), and most of them were round or irregular. In the treatments with 3.91–15.63 μg/mL anthocyanins, the distribution of the cells became loose, and a few cells were reduced in volume and were in a floating state. In the treatments with 31.25–62.5 μg/mL anthocyanins, more than half of the cells survived, some cells were blurred, and their nuclear membranes were broken. In the treatments with 125–500 μg/mL anthocyanins, it was clear that with increasing anthocyanin concentration, the number of cells decreased sharply, the cytoskeleton disintegrated, and the nucleoplasm became condensed. In the treatments with 1000–2000 μg/mL anthocyanins, only a few cells survived, and vacuole atrophy was evident. In the treatment with different concentrations of anthocyanin, the changes in BGC-803 cells were consistent with the morphological characteristics of apoptosis, which proved that the separated samples of *Malus* ‘Royalty’ fruits could induce the apoptosis of gastric cancer cells.

#### 3.4.3. The Effects of Different Concentrations of Enriched Anthocyanins Fraction on BGC-803 Cell Apoptosis

**Figure 7:**
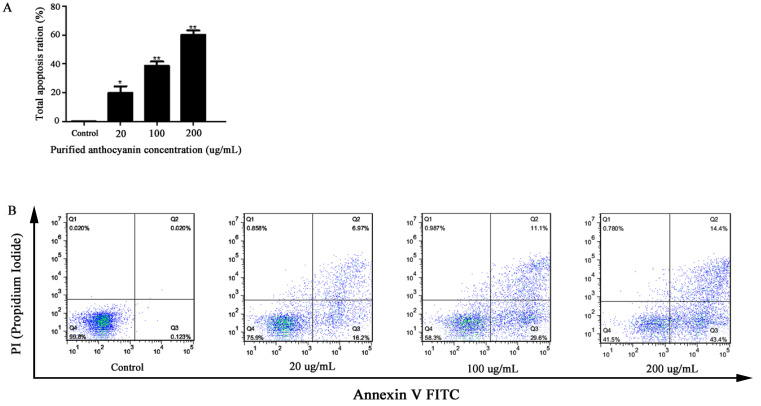
Apoptosis effects of different concentrations of the enriched anthocyanins fraction on BGC-803 cells for 48 h (0, 20, 100, 200 μg/mL). (**A**): Apoptosis results of the statistical analysis of flow cytometry detection; *: Significant difference (*p* < 0.05); ** Highly significant difference (*p* < 0.01); (**B**): The treated cells were subjected to flow cytometric analysis after PI (Propidium Iodide) and Annexin V staining.

This experiment was performed to clarify the inhibitory effect of the enriched anthocyanin fraction on BGC-803 cell apoptosis by the PI/Annexin V-EGFP double staining method. With the increase in anthocyanin purification concentration, the numbers of early, middle, and late apoptotic cells in quadrants I and IV gradually increased, while the number of necrotic cells in quadrant II did not change (Figure 7). The apoptotic rate of BGC-803 cells was 0.189 ± 0.064% in the untreated control. After exposure to different anthocyanins (20, 100, and 200 µg/mL) for 48 h, the apoptotic rates were 19.87 ± 4.67%, 28.55 ± 3.04%, and 60.01 ± 3.11%, respectively. Compared with the control, the apoptosis rates of the cells treated with anthocyanin increased by 19.68%, 28.36%, and 59.82%, respectively. The results illustrated that anthocyanins induced cell apoptosis in a dose-dependent manner.

#### 3.4.4. The Effects of Anthocyanins at Different Concentrations on Protein in BGC-803 Cells

**Figure 8:**
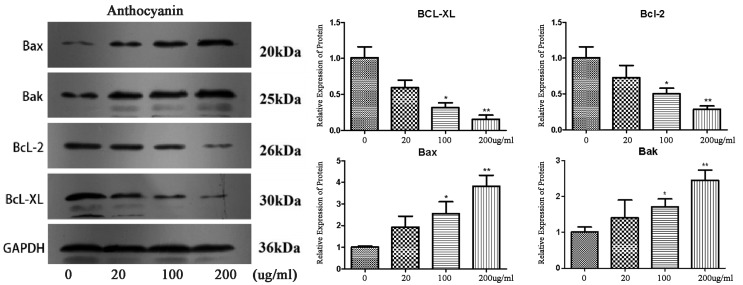
Expression levels of apoptosis-related proteins (Bax, Bak, Bcl-2, and Bcl-xl) by western blotting. GAPDH was treated as a control. The results are expressed as the mean ± SD (*n* = 3). Different asterisks indicate a significant difference at *p* < 0.05.

To explore the mechanism of BGC-803 cell apoptosis induced by the enriched anthocyanin fraction from *Malus* ‘Royalty’ fruits, Western blotting was used to detect the expression of the protein in BGC-803 cells induced by purified anthocyanins. B-cell lymphoma (BCl) is a family of key apoptotic proteins including anti-apoptotic proteins (Bcl-2 and Bcl-xL) and pro-apoptotic proteins (Bax and Bak). A higher concentration of anthocyanins had a more significant effect on the protein expression level (Figure 8).

Compared with the reference protein GAPDH, BGC-803 cells were treated with different concentrations of the enriched anthocyanin fraction (0, 20, 100, 200 μg/mL) for 48 h, and the expression signals of the antiapoptotic proteins Bcl-xL and Bcl-2 decreased gradually. A 4.25-fold decrease in the Bcl-xL/Bcl-2 ratio was achieved in gastric cancer cells compared with the untreated control upon anthocyanidin treatment. In contrast, the expression levels of the apoptotic proteins Bax and Bak gradually increased. The Bax expression level was 3.5 times higher than that of the untreated control upon anthocyanidin treatment, indicating that the expression of Bcl-xL and Bcl-2 protein was downregulated, and the expression levels of the Bax and Bak proteins were upregulated. The results showed that with the increase in *Malus* ‘Royalty’ fruit anthocyanin concentration in the medium, the expression levels of the anti-apoptotic proteins Bcl-2 and Bcl-xL decreased, and the expression levels of the pro-apoptotic proteins Bax and Bak increased, which coordinated to regulate BGC-803 cell apoptosis.

## 4. Discussion

*Malus* ‘Royalty’ fruits are red, have a high anthocyanin content, and are a new plant resource for anthocyanin extraction. Anthocyanin production decreased with increasing temperature (Figure 2A). A study on the effect of temperature on the yield of anthocyanins from blueberries showed similar trends [25]. The reason might be that the increase in temperature leads to the degradation of anthocyanins and structure destruction, which influences the extraction effect [26]. With the ethanol content increasing from 20% to 60%, the extraction yield increased, but when the ethanol content increased to 100%, instead, the anthocyanin yield decreased (Figure 2C). This phenomenon is related to the hydrophilicity of anthocyanins. As the proportion of water in the extraction solvent decreased, the hydrophilic anthocyanins became harder to dissolve. According to the report, the extraction of hydrophilic anthocyanins requires the presence of a certain amount of water [27]. The yield of anthocyanins extracted increased as the solid–liquid ratio increased from 1:2 g/mL to 1:5 g/mL and then slightly decreased when the ratio increased to 1:6 g/mL. Zhang et al. [28] observed that fixing the content of the extract and further increasing the amount of extraction solvent increased the yield of anthocyanins before an eventual decrease. Upon testing six single factors that affect the content of anthocyanin extracts, the results showed that temperature, ethanol content, and solid–liquid ratio content had significant effects on the anthocyanin yield.

Through macroporous resin column chromatography and dynamic elution experiments, the separation of the anthocyanin sample obtained was 69.4% by the area normalization method. The chromatographic profile of the separated sample at 525 nm was determined to be cyanidin-3-galactoside, a result that was consistent with a previous study showing that anthocyanins in *Malus* ‘Royalty’ fruits are mainly cyanidin-3-galactoside [29]. Compared with research that reported an anthocyanin extraction purity of 42.91% from *Lonicera caerulea* ‘Beilei’ fruits purified by macroporous resin [30], the purity of the anthocyanins obtained by the separation of *Malus* ‘Royalty’ fruits was higher. These results indicate that red *Malus* ‘Royalty’ fruits are a super raw material for the extraction of anthocyanins.

The anthocyanins of *Malus* ‘Royalty’ fruits had an IC_50_ value of 105.5 μg/mL; in brief, high concentrations of anthocyanins had powerful inhibitory effects on the proliferation of BGC-803 cells, which was consistent with previous research on the inhibitory effects of anthocyanins on colon cancer and cervical cancer cells [31,32]. There is increasing evidence that anthocyanins induce apoptosis in tumor cells. For example, the anthocyanins extracted from blueberry could promote apoptosis in HepG-2 cells [33]. Anthocyanins extracted from purple sweet potato can induce apoptotic cell death in bladder cancer cells, which is related to inhibiting the activation of signaling pathways [34]. Moreover, this induction is often dose-dependent—higher concentrations of anthocyanins have a more powerful inhibitory effect on the growth of cancer cells [32]. After anthocyanin treatment, the morphological changes in BGC-803 cells were consistent with the morphological characteristics of apoptotic cells. That is, the cell shrinkage and deformation, cytoplasm concentration, and cytoskeleton disintegration observed indicate that the enriched anthocyanin fraction could induce the apoptosis of gastric cancer cells.

Bcl (B-cell lymphoma) is a necessary apoptotic protein family involved in multiple caspase pathways. It consists of anti-apoptotic proteins (Bcl-2, Bcl-xL) and pro-apoptotic proteins (Bax, Bak) [35]. Through these pathways, the apoptosis of cytoplasmic proteins is regulated, the permeability of the outer mitochondrial membrane is changed, and the activation of proteases is effectively inhibited; therefore, external pathogens can be resisted [36]. Previous studies have proven that a decrease in the ratio of Bcl-2/Bax induces a positive effect/significance for tumor treatment. Shih et al. [37] found that after anthocyanin treatment, the ratio of Bax/Bcl-2 in gastric cancer cells increased by 1.6 times compared with the untreated blank group. The mechanism was through the upregulated expression levels of pro-apoptotic proteins and downregulated the expression levels of apoptosis-inhibitory proteins, thereby accelerating the apoptosis of cancer cells.

Western blotting was applied to detect the levels of apoptosis-related proteins in BGC-803 cells induced by anthocyanins to explore the mechanisms of apoptosis. One of the essential ways that the enriched anthocyanin fraction from *Malus* ‘Royalty’ fruits induce apoptosis of BGC-803 gastric cells might be through the inhibition of cancer cell proliferation by the regulation of the mitochondrial pathway. In summary, these results suggest that the enriched anthocyanin fraction from the *Malus* ‘Royalty’ fruits directly induce the apoptosis, but not the necrosis of BGC-803 cells.

## 5. Conclusions

Based on the results of this study, it can be concluded that the optimal extraction conditions can be defined as follows: extraction at 30 °C, solid–liquid ratio of 1:4 (g/mL), ethanol content and formic acid content of 70% and 0.4%, respectively, the extraction time of 40 min, and ultrasonic power of 300 W. Under the above conditions, the highest yield of anthocyanin production was achieved. The AB-8 macroporous resin was a better purifying material with 60% ethanol as an adsorbent, and the adsorption-desorption equilibrium times were 6 h and 1 h, respectively. Cyanidin-3-galactoside was the main body composition of total anthocyanins separation from *Malus* ‘Royalty’ fruits. The concentration of enriched anthocyanins fraction showed a dose-dependent effect on cell inhibition, and high concentrations of anthocyanins had a significant inhibitory effect on BGC-803 cells. With the increase in *Malus* ‘Royalty’ fruit anthocyanin concentration in the medium, the expression levels of the anti-apoptotic proteins Bcl-2 and Bcl-xl decreased, and the expression levels of the pro-apoptotic proteins Bax and Bak increased, which coordinated the regulation of BGC-803 cell apoptosis.

## Data Availability

All data is contained within text.

## Supplementary Materials

The following supporting information can be downloaded at: https://www.mdpi.com/article/10.3390/molecules27134299/s1, Figure S1: Calibration curve for standard Cyanidin-3-galactoside.; Figure S2: Photometric spectrogram of the eluent during the dynamic elution of anthocyanins; Figure S3 HPLC-DAD chromatogram at 525 nm of the purified anthocyanin.

## Appendix A

**Table A1:** The Box–Behnken design and the results of the response surface methodology.

Runs	Factors			Response Value
	A: Extraction Temperature (°C)	B: Ethanol Content (%)	C: Solid–Liquid Ratio (g/mL)	Anthocyanin Yield (mg/g)
1	20	60	1:5	1.6745 ± 0.0110
2	20	60	1:5	1.5615 ± 0.0131
3	30	50	1:5	1.5955 ± 0.0024
4	20	50	1:4	1.4948 ± 0.0173
5	20	60	1:5	1.5875 ± 0.0001
6	10	70	1:5	1.8895 ± 0.0278
7	20	70	1:4	1.9220 ± 0.0110
8	10	60	1:6	2.1174 ± 0.0250
9	30	60	1:6	1.9956 ± 0.0146
10	30	60	1:4	1.9424 ± 0.0049
11	20	60	1:5	1.7305 ± 0.0062
12	10	60	1:4	1.6512 ± 0.0092
13	30	70	1:5	1.9535 ± 0.0089
14	20	50	1:6	1.7466 ± 0.0045
15	20	60	1:5	1.5155 ± 0.0027
16	20	70	1:6	2.1264 ± 0.0026
17	10	50	1:5	1.4695 ± 0.0089

Note: Each experiment was repeated thrice, and the experimental results are presented as mean ± SD.

**Table A2:** Analysis of variance (ANOVA) for the response surface quadratic model.

Source	Sum of Squares	DF	Mean Square	F Value	Prob > F	Significant
Model	0.73	9	0.081	17.57	0.0005	**
A	0.024	1	0.024	5.18	0.0569	
B	0.31	1	0.31	67.55	<0.0001	***
C	0.12	1	0.12	25.79	0.0014	**
AB	9.610 × 10^−4^	1	9.610 × 10^−4^	0.21	0.6619	
AC	0.041	1	0.041	8.96	0.0201	*
BC	6.213 × 10^−4^	1	6.213 × 10^−4^	0.13	0.7245	
A^2^	0.050	1	0.050	10.77	0.0134	*
B^2^	8.338 × 10^−5^	1	8.338 × 10^−5^	0.018	0.8968	
C^2^	0.22	1	0.22	46.86	0.0002	**
Residual	0.032	7	4.613 × 10^−^³			
Lack of Fit	1.900 × 10^−^³	3	6.332 × 10^−4^	0.083	0.9656	N
Pure Error	0.03	4	7.598 × 10^−^³			
Cor Total	0.76	16				
R^2^ = 0.958	R^2^_Adj_ = 0.903	Adeq Precision = 12.51				

Note: A—temperature; B—ethanol content; C—solid–liquid ratio; DF—degree of freedom; *: Significant difference (*p* < 0.05); ** Highly significant difference (*p* < 0.01); *** The most significant difference (*p* < 0.001); N—not significant.
