# Supplementary material for: Ultrasound-Assisted Extraction of Anthocyanins from Malus ‘Royalty’ Fruits: Optimization, Separation, and Antitumor Activity
Source: Molecules. 2022 Jul 4;27(13):4299. doi: 10.3390/molecules27134299 (PMC9268470; doi:10.3390/molecules27134299)
Supplement: Supplementary file 1 [file molecules-27-04299-s001.zip › molecules-1795617-supplementary.pdf]

molecules

MDPI

Article

Ultrasound-Assisted Extraction of Anthocyanins from *Malus* 'Royalty' Fruits: Optimization, Separation, and Antitumor Activity

Yixin Liu [1,†], Yuheng Zhao [1,†], Yue Zhuo [1], Yuwen Li [2], Jiaxin Meng [1], Yilin Wang [1] and Houhua Li [1,*]

1 College of Landscape Architecture and Art, Northwest A & F University, Yangling 712100, China; lyx13426325830@126.com (Y.L.); wn15553803032@163.com (Yuheng Zhao); zhuoyuegongzuo@163.com (Yue Zhuo); mengjiaxin26@163.com (J.M.); wangyilin97417@163.com (Y.W.)
2 Shanghai United International School, Gubei Secondary Campus, Shanghai 201103, China; ellaliyuwen@icloud.com
* Correspondence: lihouhua@nwafu.edu.cn
† Yixin Liu and Yuheng Zhao contributed equally to this work.

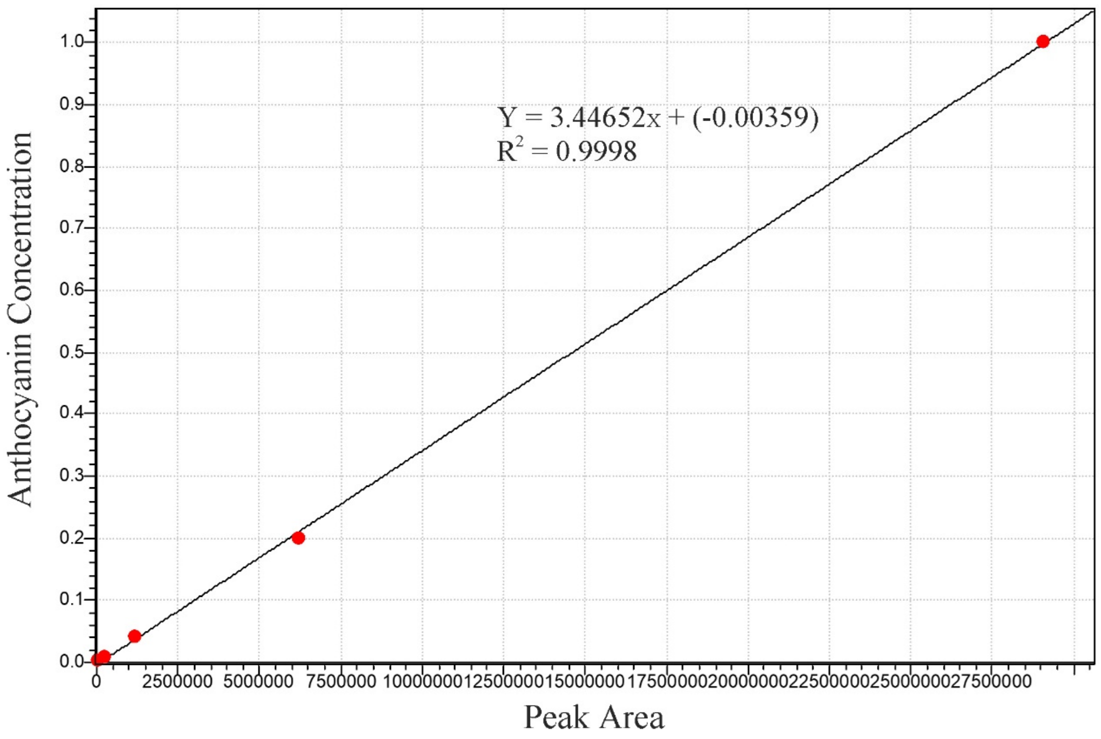

Figure S1. Calibration curve for standard Cyanidin-3-galactoside.

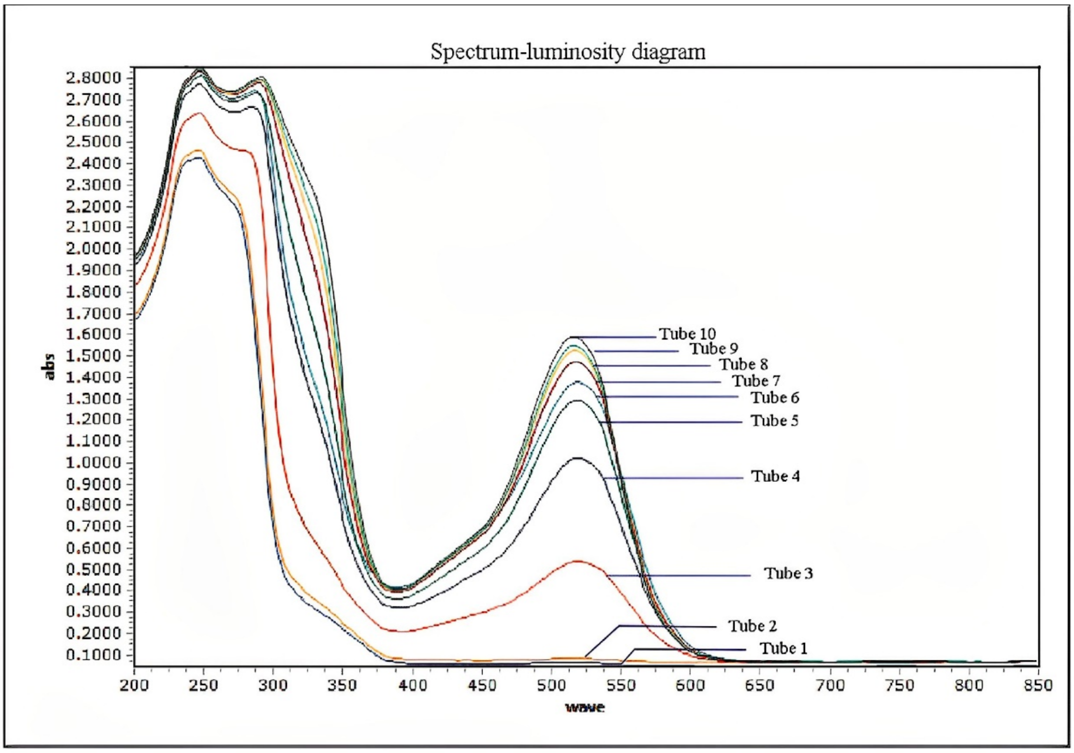

Figure S2. Photometric spectrogram of the eluent during the dynamic elution of anthocyanins.

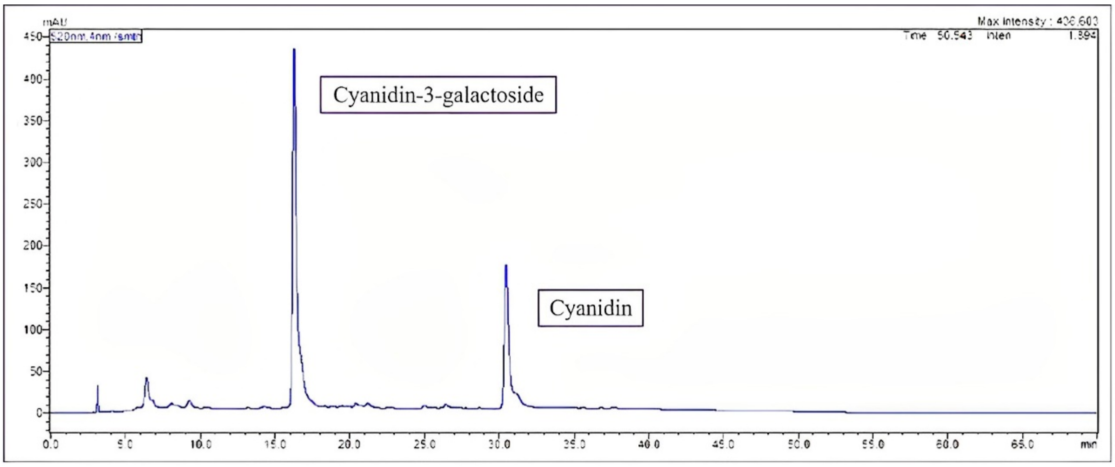

Figure S3. HPLC-DAD chromatogram at 525 nm of the purified anthocyanin.
